# The Role of Trans-Oesophageal Echocardiography in the Interventional Cardiology of Adult Congenital Heart Diseases

**DOI:** 10.3390/jcm14041049

**Published:** 2025-02-07

**Authors:** Mario Giordano, Giancarlo Scognamiglio, Gianpiero Gaio, Raffaella Marzullo, Michela Palma, Rosaria Barracano, Flavia Fusco, Nunzia Borrelli, Simona Sperlongano, Giovanni Cimmino, Maria Giovanna Russo, Berardo Sarubbi

**Affiliations:** 1 Paediatric Cardiology Division, University of Campania “Luigi Vanvitelli”, Monaldi Hospital, AORN Ospedali dei Colli, 80131 Naples, Italy; gianpierogaio@hotmail.com (G.G.); ra.marzullo@gmail.com (R.M.); mgiovannarusso@gmail.com (M.G.R.); 2 Adult Congenital Heart Disease Division, Monaldi Hospital, AORN Ospedali dei Colli, 80131 Naples, Italy; giancascognamiglio@gmail.com (G.S.); plmichela@hotmail.it (M.P.); rosariabarracano@libero.it (R.B.); flaviafusco90@gmail.com (F.F.); nunziaborrelli16@gmail.com (N.B.); berardo.sarubbi@virgilio.it (B.S.); 3 Cardiology Division, University of Campania “Luigi Vanvitelli”, 80138 Naples, Italy; sperlongano.simona@gmail.com (S.S.); giovannicimmino76@yahoo.it (G.C.)

**Keywords:** trans-oesophageal echocardiography, interventional cardiology, adult congenital heart disease, catheterization

## Abstract

Advances in interventional cardiology have significantly broadened the range of congenital heart diseases (CHDs) amenable to trans-catheter interventions. Trans-oesophageal echocardiography (TOE) plays a pivotal role as a procedural guide in several percutaneous treatments. Enhanced imaging modalities and technological innovations in echocardiography have refined the precision and applicability of these approaches. This review explores the role, impact, and advancements of TOE in trans-catheter treatments for adult CHDs, including both common procedures (e.g., atrial septal defect closure, ventricular septal defect closure) and less frequent interventions (e.g., Mustard/Senning baffle leak closure, Fontan conduit fenestration, ruptured sinus of Valsalva embolization).

## 1. Introduction

Interventional cardiology for adult congenital heart diseases (ACHDs) has transformed the therapeutic landscape for these patients, improving outcomes and long-term prognoses. Procedures such as atrial septal defect (ASD) and ventricular septal defect (VSD) closure, management of atrial switch operation baffle leaks, Fontan fenestration, and ruptured sinus of Valsalva embolization heavily rely on trans-oesophageal echocardiography (TOE) for both pre-procedural assessment and intra-procedural guidance.

**Figure 1:**
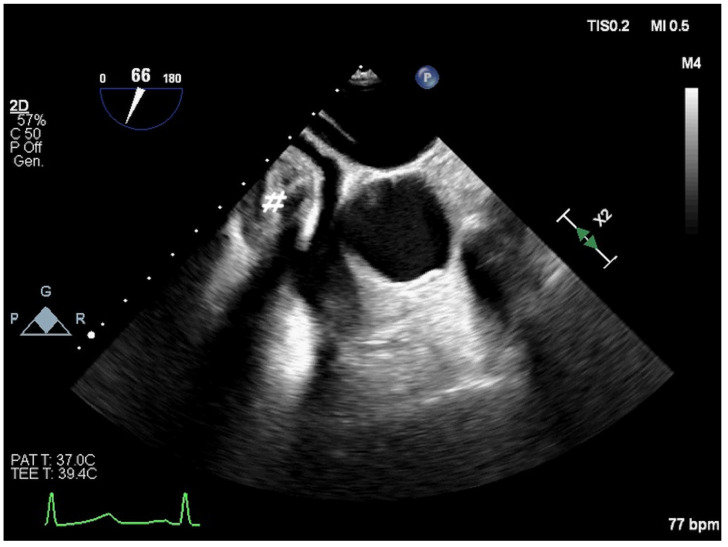
Two-dimensional TOE. Mid-oesophageal view. Extracardiac mass (pseudo-aneurysm, probably) (#) with “ab-extriseco” right atrial compression.

During the pre-operative phase, TOE evaluates the feasibility of percutaneous treatments and aids in meticulous procedural planning, including vascular access and the selection of guidewires, catheters, and devices. TOE is instrumental in identifying contraindications such as intra-cardiac thrombi, infective endocarditis, and cardiac anomalies requiring surgical correction (e.g., anomalous pulmonary vein drainage, significant valvular dysfunction, or aortic root dilatation). Moreover, TOE can identify extra-cardiac abnormalities, such as masses causing cardiac compression, necessitating advanced imaging (e.g., angio-CT, angio-MRI) (Figure 1).

Intra-procedurally, TOE provides real-time imaging, enabling precise execution of each procedural step to minimize complications. Known as “the eye of the interventional cardiologist,” TOE remains the most adopted ultrasound guidance, despite emerging alternatives such as intracardiac echocardiography. A recent metanalysis showed no differences between TOE and trans-thoracic echocardiography procedural monitoring regarding the rates of success and residual shunt in both atrial and ventricular septal defect closure [1]. However, significant operator experience is necessary to avoid procedural failure and/or significant adverse events.

This review highlights the role of TOE in ACHD interventions, focusing on its contributions to anatomical evaluation, procedural planning, and intra-operative monitoring. In the building of the review, the most impacted papers about the argument were considered to achieve a detailed and complete analysis of the single topics.

## 2. Atrial Septal Defect Closure

**Pre-operative assessment.** In contemporary practice, trans-catheter device closure is considered the first-line approach for addressing “ostium secundum” ASDs. The pre-operative TOE evaluation serves multiple objectives: assessing periorificial rims, ASD size and shape, accessory ASDs or fenestrations, and interatrial septum anatomy (e.g., total length, aneurysms, double atrial septum or spiral-shaped septum).

**Figure 2:**
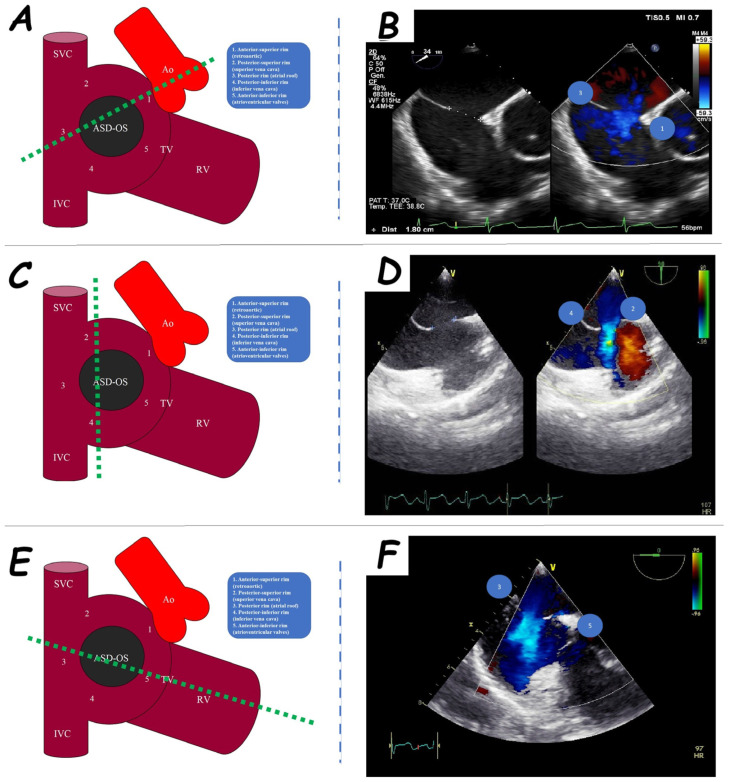
Periorificial rim assessment. Anterior-superior and posterior rims (**A**) are evaluated in 45° mid−oesophageal view (**B**), anterior−inferior and posterior rims (**C**) in 0° mid−oesophageal view (**D**), and posterior−superior and posterior−inferior rims (**E**) in 90° mid−oesophageal view (**F**). Abbreviations: Ao, aorta; ASD-OS, atrial septal defect ostium secundum; IVC, inferior vena cava; RV, right ventricle; SVC, superior vena cava.

The evaluation of periorificial rims is pivotal for trans-catheter closure. Five ASD rims are categorized: anterior-superior or retro-aortic rim (adjacent to the aortic root), posterior-superior rim (near the superior vena cava), posterior rim (close to the atrial roof and right pulmonary vein orifices), posterior-inferior rim (near the inferior vena cava), and anterior-inferior rim (proximal to the atrioventricular valves) (Figure 2). A rim may be classified as adequate (>5 mm), poor (<5 mm), or absent [2,3]. Absence or deficiency of the retro-aortic rim is relatively common and does not constitute an absolute contraindication for percutaneous closure, although it carries a higher risk of aortic erosion [4]. In contrast, deficiencies in other rims can complicate the procedure and heighten the risks of trans-catheter failure [5], cardiac erosion [6], and device embolization [7]. When posterior rim deficiency is present, the extent of the deficiency is critical in determining the feasibility of percutaneous closure [7].

**Figure 3:**
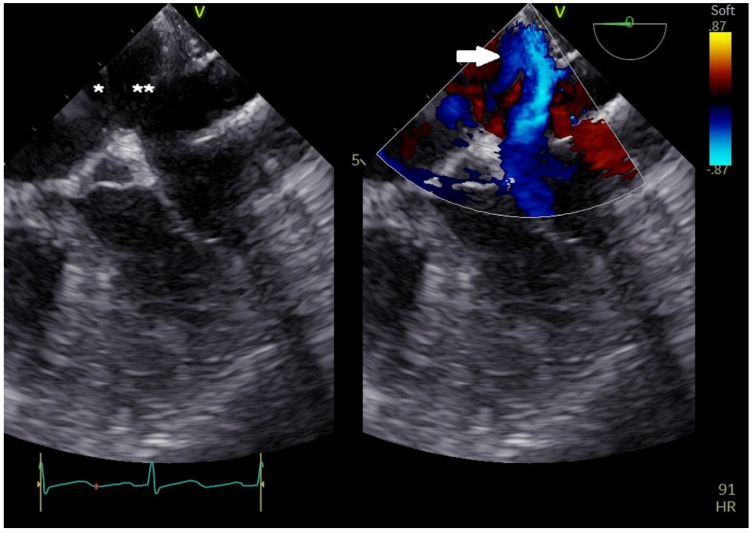
Two−dimensional TOE. Mid−oesophageal view. ASD with “octopus shape” [arrow] due to an associated double interatrial septum (right-side septum [*] and left-side septum [**]).

**Figure 4:**
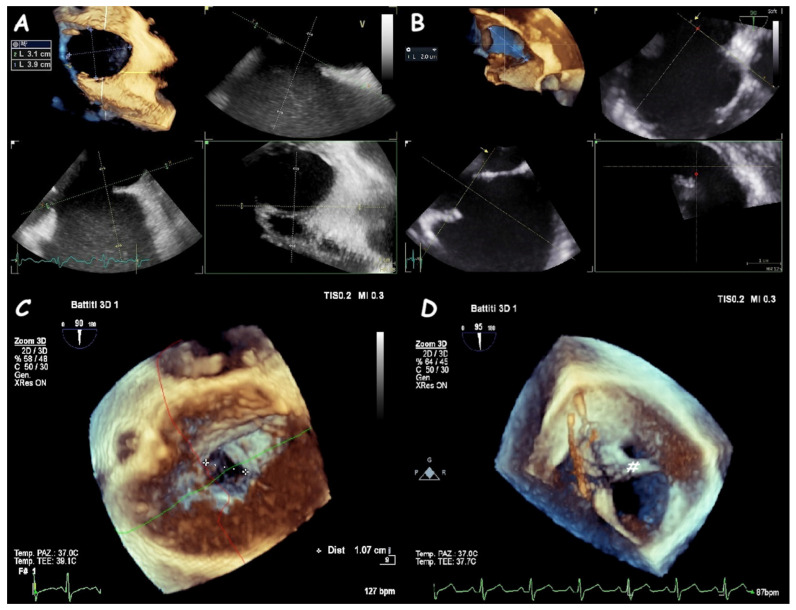
Three−dimensional TOE view of a circular-shape ASD (**A**), an oval−shape one (**B**), an irregular−shape (like a tennis racket) one (**C**), and a large ASD with an internal seeding (#) (**D**).

ASDs vary significantly in size and shape, exhibiting elliptical, round, or irregular morphologies (e.g., star- or reniform-shaped) [8]. Occasionally, a single defect with irregular borders may mimic multiple defects, manifesting a characteristic “octopus-like” appearance (Figure 3). Three-dimensional (3D) TOE provides the most precise evaluation of ASD dimensions and shapes. Consequently, 3D TOE is indispensable for identifying large ASDs with internal seedings that might appear as multiple closely situated defects on two-dimensional (2D) ultrasound (Figure 4).

**Figure 5:**
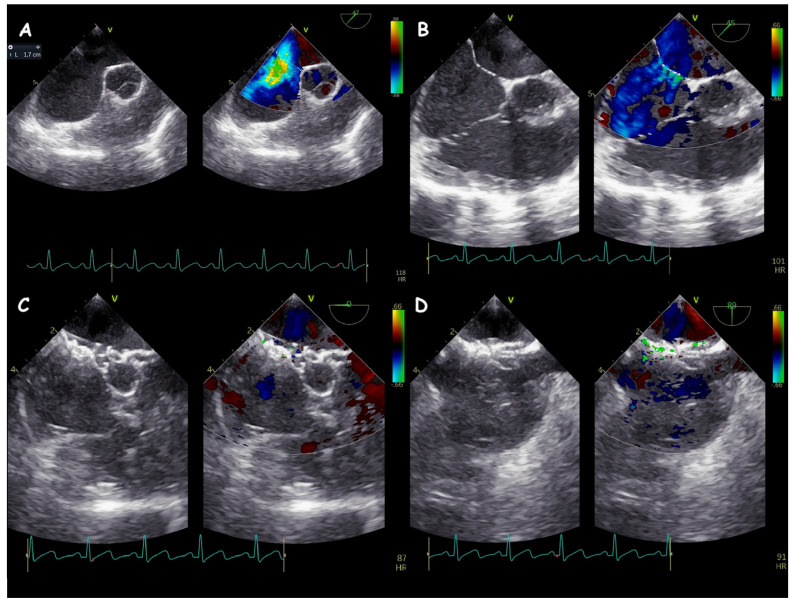
Two−dimensional TOE. Mid−oesophageal view highlights a large 17 mm ASD (**A**) with multiple accessory fenestration (**B**). A single occluder device was able to have complete closure of every interatrial shunt (**C**,**D**).

In some cases, a primary ASD may be accompanied by one or more accessory interatrial defects or fenestrations. The presence of multiple interatrial shunts poses challenges for interventional cardiologists, often necessitating the use of multiple devices for complete closure. Key factors guiding percutaneous closure include the layout of the interatrial septum and shunts, as well as the sizes and distances between shunts [9,10]. Typically, one or more smaller adjacent defects (<7 mm from the larger defect) can be addressed by placing a single device in the larger defect [11] (Figure 5).

**Figure 6:**
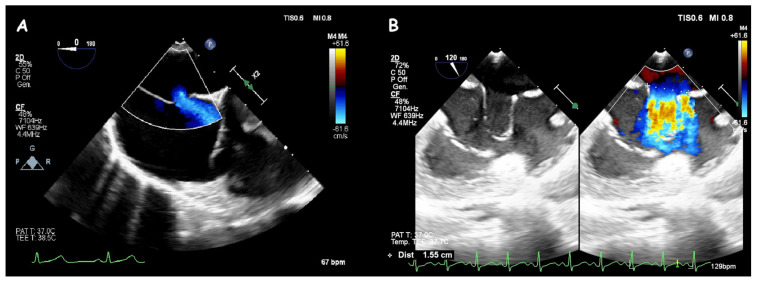
Two−dimensional TOE. Mid-oesophageal view of a hypermobile interatrial septum with a single ASD (**A**) and an aneurysmatic interatrial septum (base width 15 mm) with multiple interatrial shunts (**B**).

**Figure 7:**
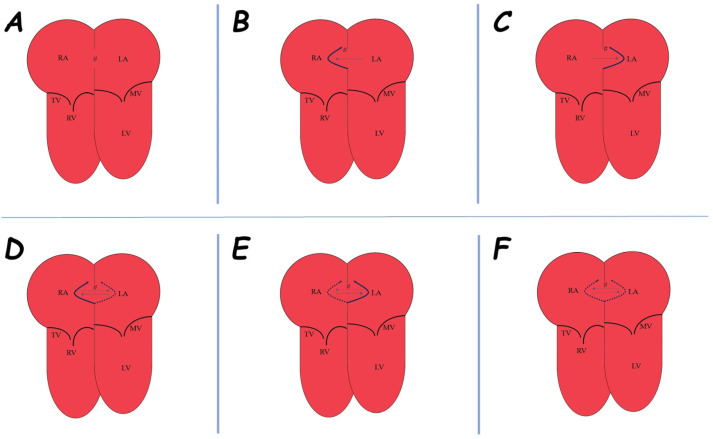
Graphic scheme of no-aneurysmatic ASD (#) (**A**) and different types of aneurysmatic interatrial septum: type 1R (**B**), type 2L (**C**), type 3RL (**D**), type 4LR (**E**), and type 5 (**F**). Abbreviations: LA, left atrium; LV, left ventricle; MV, mitral valve; RA, right atrium; RV, right ventricle; TV, tricuspid valve.

A redundant interatrial septum is classified as “aneurysmal” when its base width ≥ 15 mm and excursion > 10 mm into either atrium [12] (Figure 6). When these criteria are not fully met, the septum is termed “hypermobile.” Olivares-Reyes et al. identified five aneurysmal interatrial septum types: type 1R (dextro-convex, protruding into the right atrium), type 2L (left-convex, protruding into the left atrium), type 3RL (bidirectional waving, predominantly toward the right atrium), type 4LR (bidirectional waving, predominantly toward the left atrium), and type 5 (completely bidirectional without a prevalent excursion) [13] (Figure 7). Aneurysmal interatrial septa are frequently associated with multiple ASDs or fenestrations [10], often necessitating multiple devices. Additionally, balloon sizing of an ASD within an aneurysmal septum may reveal a larger-than-expected diameter due to over-stretching of the floppy, hypermobile septal tissue [10].

**Figure 8:**
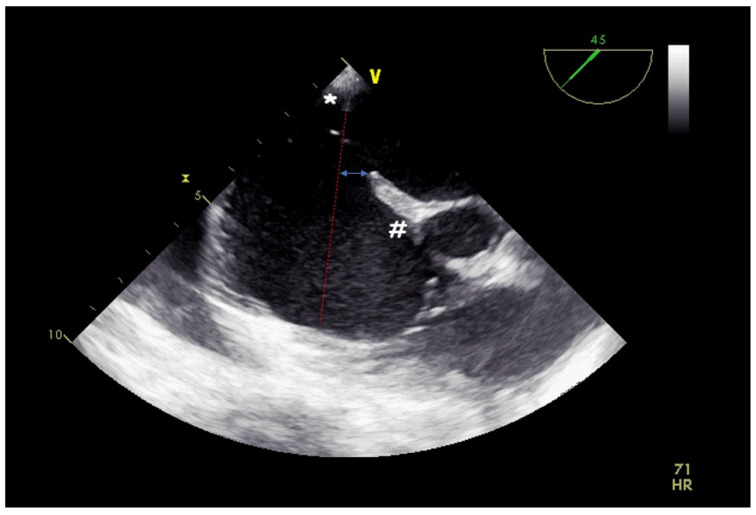
Two-dimensional TOE. Mid-oesophageal view of a central ASD with a poor floppy posterior rim (*) and a well-represented anterior-superior rim (#). The septal malalignment may be highlighted extending the plane passing through the posterior rim (dotted red line) and measuring the distance of the latter from the anterior-superior rim (double arrow blue line).

Misalignment of the septum primum toward the left atrium results in a misaligned ASD [14]. The separation distance between the septum primum and the septum secundum surfaces determines the “severity” of the malalignment [14] (Figure 8). Severe malalignment (≥10 mm separation) may increase procedural failure risk [15,16]. While trans-thoracic echocardiography may miss this feature, TOE’s high sensitivity is crucial for detecting septal malalignment. Oversized devices are often required for successful ASD closure under these circumstances [14]. Extreme septal malalignment can occasionally cause concomitant partial or total anomalous pulmonary vein drainage (with normal pulmonary vein connection), necessitating surgical intervention [17].

**Figure 9:**
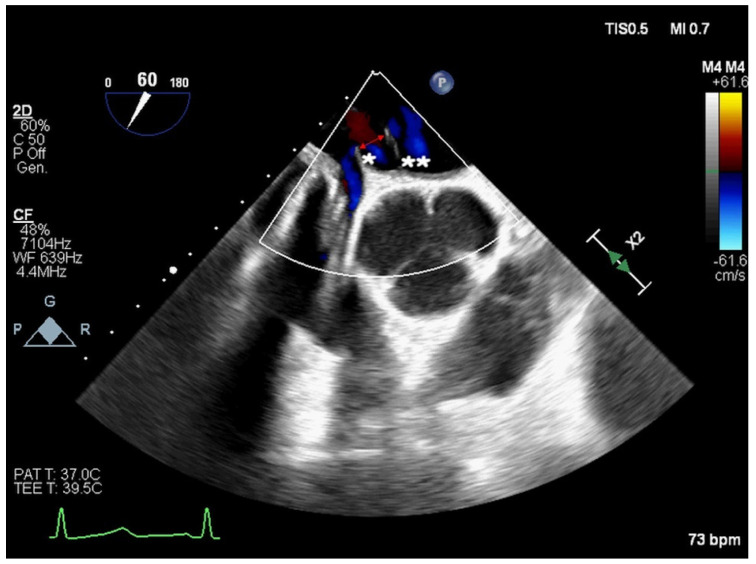
Two−dimensional TOE. Mid-oesophageal view of a double interatrial septum (i.e., spiraliform septum). The distance (red double arrow) between the right septum (*) and the left one (**) defines the width of the double septum.

Double atrial septum (DAS), or spiraliform interatrial septum, represents an anatomical variation caused by embryological misalignment of the primitive atrial septum. DAS is often associated with ASDs exhibiting wide separations between the left and right atrial rims of the defect [18]. The misaligned rims in DAS increase the risk of procedural failure or device embolization. Deploying an oversized device may mitigate these risks [16]. Accurate diagnosis of DAS, often requiring 3D TOE due to the limitations of trans-thoracic echocardiography and 2D TOE, is critical for procedural safety (Figure 9).

Identifying a redundant Eustachian valve or Chiari network is important, as these structures may impede device deployment, requiring specific technical skills for successful trans-catheter ASD closure [19].

Key features for pre-procedural assessment in trans-catheter ASD closure are summarized in the Table 1.

Pre-procedural TOE is also essential for identifying associated cardiac lesions. For instance, partial anomalous pulmonary vein connection, undetected by trans-thoracic echocardiography, might result in diastolic right ventricular overload being incorrectly attributed solely to the ASD. TOE facilitates comprehensive diagnosis, guiding patients toward appropriate surgical repair.

**Intra-procedural monitoring.** TOE plays a pivotal role during percutaneous ASD closure. Balloon sizing of the ASD is monitored to confirm complete defect occlusion and rule out residual interatrial shunts. A residual shunt during balloon occlusion may result from either incomplete balloon inflation (peri-balloon residual shunt) or accessory fenestrations [20]. Distinguishing between these mechanisms is crucial; the former risks selecting an undersized device with increased embolization potential, whereas the latter scenario often allows closure of adjacent fenestrations by the prosthesis (especially if the fenestration is <7 mm from the main ASD) [11].

**Figure 10:**
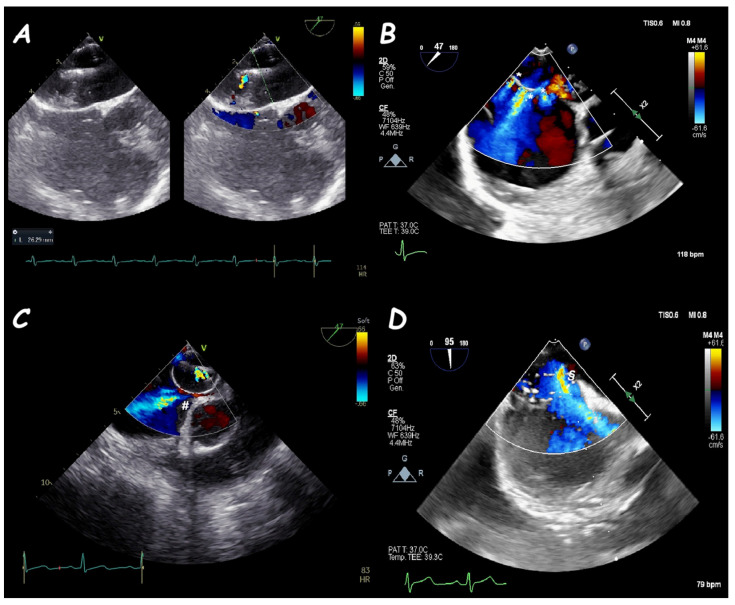
Two−dimensional TOE. Mid−oesophageal view of the following: an optimal static balloon interrogation without residual shunt (**A**), a residual shunt due to three accessory fenestrations (*) during a static balloon interrogation (**B**), a residual shunt due to under-inflation of the balloon (#) due to dynamic balloon sizing (**C**), a large ASD with internal seeding with a large residual shunt (arrow) due to the lack of seeding rupture during static balloon sizing (**D**).

In ASDs with internal seedings, balloon sizing aims to rupture the seeding, ensuring an adequate device fit and reducing embolization risk (Figure 10). Rarely, atrial septal laceration during balloon sizing may occur, resulting in interatrial septum hematoma, which often necessitates surgical repair [21,22].

Narimani et al. recently demonstrated a strong correlation between ASD perimeter measurements obtained via 3D TOE and balloon-sizing diameters, irrespective of ASD shape (circular or oval) [23].

**Figure 11:**
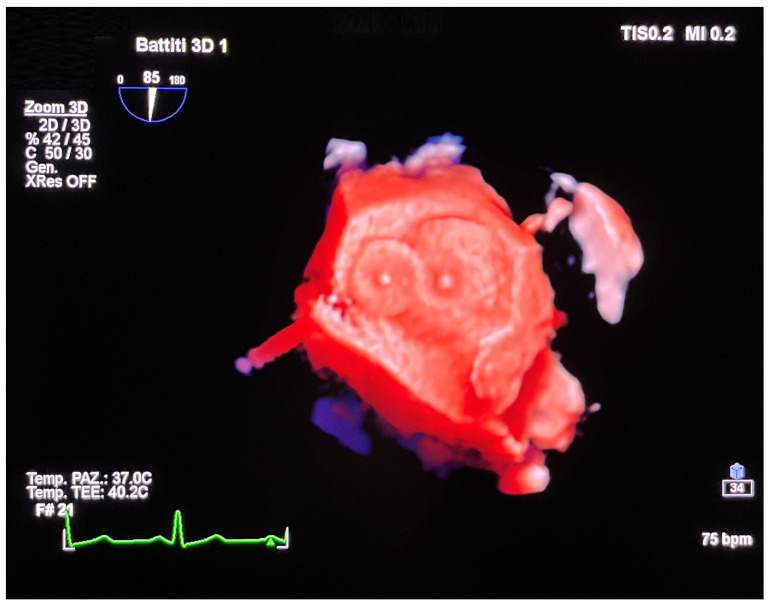
Three-dimensional TOE. Left atrial view of two occluder devices in partial overlap.

In multi-fenestrated interatrial septa, TOE clarifies which fenestration has been crossed by the guidewire or catheter. Crossing the largest or central fenestration/ASD is recommended to optimize closure and minimize the number of devices required [9,10]. In the cases requiring multiple devices, the TOE should check the relationship between the prosthesis. Frequently, a close relationship with device overlapping is frequent. In this subset, it is recommended to have a “sandwich relationship” with the smaller device included by the larger one (left disc occluder #1, left disc occluder #2, right disc occluder #2, right disc occluder #1) or an “interleaved relationship” with a disc intersection between the prosthesis (left disc occluder #1, left disc occluder #2, right disc occluder #1, right disc occluder #2) [24,25]. The 3D TOE increases the angiographic and the 2D ultrasound capability to evaluate the device relationship (Figure 11). Nowadays, personalized 3D printing and echo-guided procedures help to improve procedural planning, increasing the chance of achieving complete closure of all interatrial shunts with a single device [26].

**Figure 12:**
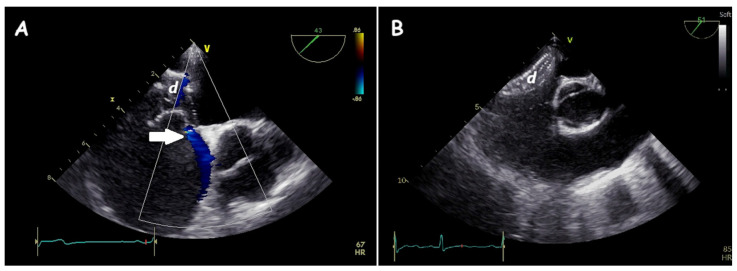
Two−dimensional TOE. Mid−oesophageal view of a partial prolapse of the left disc of the device (d) with mild residual shunt (arrow) (**A**) and complete prolapse of the device (d) in the right atrium (**B**) during “push−and−pull” maneuver. In both cases, the device was substituted witha larger one.

Following device implantation, TOE assesses prosthesis deployment, ruling out interference with periorificial structures (e.g., pulmonary/systemic veins, atrioventricular valves, coronary sinus ostium, atrial roof, or aortic root). Adequate anchoring is verified via a “push-and-pull maneuver” to mitigate embolization risks (Figure 12). The relationship between the device and the aortic root should always be monitored. Intermittent contact, splaying, and protrusion or a motion of the device towards the aortic root are predictive of a higher risk of device-related erosion [27].

TOE also detects potential complications, including pericardial effusion (or tamponade), atrioventricular valve perforation, aortic root deformation, and thrombosis of guidewire, catheters, or device discs. Device deformation, such as cobra-shaped distortion, may occur during or after implantation.

## 3. Ventricular Septal Defect Closure

**Pre-operative assessment.** Trans-catheter closure of VSDs is an emerging procedure in interventional cardiology, particularly for ACHDs. Currently, most VSDs require surgical closure; however, muscular VSDs and peri-membranous VSDs with adequate peri-orificial rims (greater than 2 mm by the atrioventricular, aortic, and pulmonary valves) may be suitable for percutaneous closure.

During the pre-operative assessment, TOE serves multiple purposes, including VSD topographic classification (peri-membranous vs. muscular VSD; inlet, outlet, or trabecular VSD; malalignment VSD; Gerbode VSD), as well as evaluating size and shape (circular, oval, or irregular) and number (single, double, or multiple VSDs). It also helps in determining the relationship with peri-orificial structures (aortic and pulmonary valves, tricuspid and mitral valves, atrioventricular chords), accessory features (e.g., VSD with an aneurysmal pouch or multiple interventricular septum communications), and associated lesions (e.g., aortic valve dysfunction, accessory VSDs, valvular straddling and/or overriding, thrombus, or vegetation).

The International Society for Nomenclature of Paediatric and Congenital Heart Disease (ISNPCHD) [28] classifies peri-membranous VSDs into central peri-membranous VSD, inlet peri-membranous VSD (with or without atrioventricular valve malalignment), and outlet peri-membranous VSD (with or without anterior or posterior malalignment of the outlet septum). An outlet VSD close to the pulmonary valve, known as doubly committed, is characterized by partial or complete absence of the infundibular septum. Direct or indirect communication between the left ventricle and the right atrium is termed Gerbode VSD. Post-surgical residual shunts may also be eligible for percutaneous treatment.

**Figure 13:**
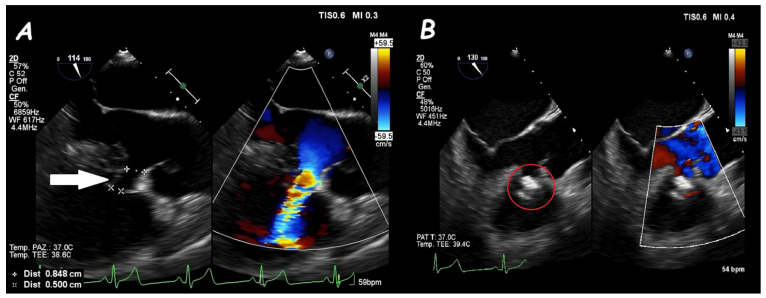
Two−dimensional TOE. Mid−esophageal view of a peri-membranous VSD within a sub-aortic aneurysm (arrow) (**A**) that underwent device (red circle) closure without residual shunt (**B**).

TOE is indispensable for identifying VSD features. A minimum distance of 2 mm from the aortic valves is usually required for percutaneous closure [29]. The presence of an aneurysmal pouch is favorable, as it facilitates optimal device deployment (positioned away from the aortic valve) [30] (Figure 13). A 3D TOE assessment provides comprehensive characterization of the ventricular aneurysm and predicts the feasibility of percutaneous closure [31]. Conversely, Laubry–Pezzi syndrome (aortic regurgitation due to cusp prolapse) or atrioventricular chord attachment on the VSD “crista” are contraindications to device closure [30]. When aortic regurgitation is present, TOE is crucial for identifying the mechanism of regurgitation, as conditions other than Laubry–Pezzi syndrome (e.g., aortic annulus dilatation or cusp fissuration) are not contraindications for trans-catheter closure.

According to ISNPCHD [28], muscular VSDs are categorized as inlet, outlet, or trabecular. Trabecular muscular VSDs may be mid-septal, apical, postero-inferior, antero-superior, or multiple (Swiss cheese septum). An unusual variant, serpiginous tunneled muscular VSD, is characterized by one or more left-side orifices, a branched interventricular tunnel, and one or more right-side orifices [32]. Mid-apical single muscular VSDs may appear as multiple VSDs due to septomarginal trabecula interference, resulting in shunts with different directions (to the apex and inlet). Pre-operative evaluation of trabecular VSD positioning is critical, as vascular access depends on VSD localization (e.g., jugular vein approach for apical VSDs and femoral vein approach for mid-septal VSDs).

Trans-catheter closure of post-surgical VSD residual shunts is feasible. Patch dehiscence, commonly located in the posterior-inferior and posterior-superior quadrants (near conductive tissue), is the primary cause of significant residual shunts [33]. Patch fibrosis often provides the necessary periorificial rims for device deployment.

**Figure 14:**
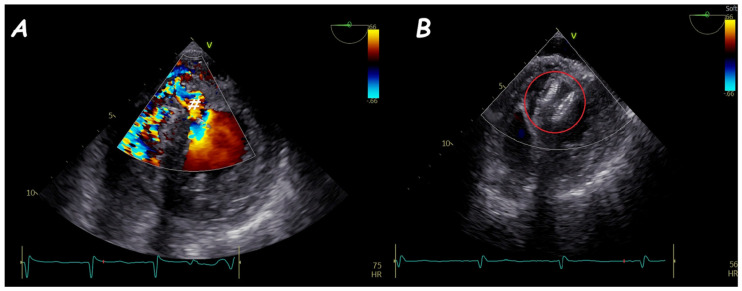
Two−dimensional TOE. Trans−gastric view of an apical post−MI VSD (#) (**A**) that underwent device (red circle) closure without residual shunt (**B**).

Post-myocardial infarction (post-MI) VSD represents a severe complication of acute coronary syndrome. In the acute phase (first two weeks), post-MI VSDs have fragile, necrotic rims due to coagulation necrosis and enzymatic degradation by neutrophils. In the chronic phase (3–4 weeks post-infarction), a firm scar consolidates the VSD rims, enabling percutaneous closure [33]. This avoids surgical intervention in high-risk patients (Figure 14). Post-MI VSDs typically have irregular shapes and are located in the posterior wall of the apical septum, although localization depends on the infarction area [34]. Three-dimensional TOE is essential to detect irregular shapes and fragile rims, aiding in optimal procedural planning [35].

Pre-operative TOE also identifies concomitant causes of left ventricular volume overload (e.g., severe aortic regurgitation, patent ductus arteriosus, aorto-pulmonary window) and associated congenital heart diseases (CHDs).

Key features for pre-procedural assessment in trans-catheter VSD closure are summarized in the Table 2.

**Intra-procedural monitoring.** Intra-procedural TOE is critical for guiding VSD closure. It monitors VSD crossing and arterial-venous loop formation. After VSD crossing, guidewires and catheters may pass through ventricular chords, potentially compromising device deployment. TOE detects such abnormal crossings and assesses guidewire interference with AV valve function and sub-valvular structures, such as chordal rupture causing flail tricuspid leaflets [36]. If interference occurs, re-crossing the VSD is recommended to prevent sub-valvular damage.

**Figure 15:**
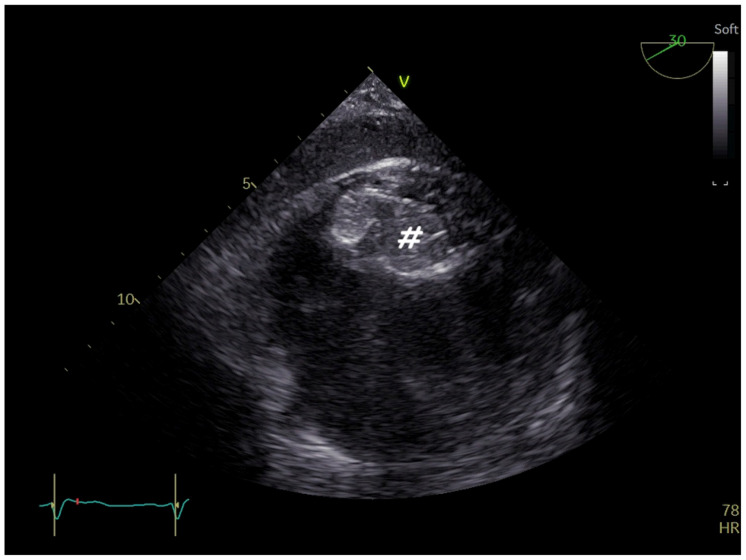
Two-dimensional TOE. Trans-gastric view of static balloon sizing (#) of an apical post-MI VSD.

Balloon VSD stretching is generally not recommended. Device sizing relies on TOE measurements, particularly for oval or irregular VSDs, to avoid underestimating defects and selecting a too-small device [37]. Balloon sizing may be used for post-MI VSDs to stretch fragile rims and prevent prosthesis embolization. Static balloon interrogation profiles irregular VSD edges and confirms complete defect occlusion without residual shunts or accessory VSDs [38] (Figure 15).

After device deployment, TOE ensures complete VSD closure without residual shunts. It rules out interference of the left disc with the aortic (outlet VSDs) or mitral (inlet VSDs) valves and interference of the right disc with the tricuspid valve (outlet and inlet VSDs). In muscular VSDs, interference with septomarginal trabeculae should also be excluded. A single left-disc device (e.g., Amplatzer Duct Occluder type I [Abbott, Plymouth, MN, USA] or Occlutech PDA Occluder [Occlutech International AB, Helsingborg, Sweden]) is a viable alternative in cases of right-disc interference with the tricuspid valve [39].

In direct Gerbode VSDs, the right disc is placed in the right atrium near the tricuspid septal leaflet, risking leaflet perforation. Novel multifunctional occluder devices are softer, reducing this risk [40].

Peri-prosthetic residual shunts must be avoided as they are associated with hemolysis and persistent left ventricular volume overload [41]. Early intra-prosthetic residual shunts are common but may cause post-operative hemolysis due to high biventricular pressure gradients [42].

Potential procedure-related complications include pericardial effusion, atrioventricular valve perforation, thrombosis (guidewire, catheters, or device discs), device malposition, and embolization.

## 4. Leak Closure of Atrial Switch Operation Baffles

**Pre-operative assessment.** Historically, the atrial switch operation (e.g., Mustard or Senning procedure using inter-atrial baffles) was the most commonly performed surgical correction for transposition of the great arteries (TGA) [43]. Today, this technique has been replaced by the arterial switch operation. However, many adult patients with repaired TGA remain under follow-up care.

The primary complications of inter-atrial baffles include significant stenosis or leakage, which may lead to inter-atrial shunting or paradoxical embolization. Trans-catheter dilatation of baffle stenosis is primarily a fluoroscopy-guided procedure, although concurrent TOE monitoring can be useful. Conversely, trans-catheter closure of baffle leaks typically involves both fluoroscopic and TOE guidance.

Pre-operative evaluation of baffle leakage is critical for procedural planning. TOE aims to assess the following: the number of leaks (one, two, or more), their size, localization (systemic or pulmonary baffle; anterior, superior, posterior, or inferior position), shape (circular, oval, linear, irregular), the amount and direction of the shunt (left-to-right, bidirectional, or right-to-left), and the relationship of the leaks to adjacent structures (e.g., superior or inferior vena cava, pulmonary veins, coronary sinus, atrioventricular valve).

**Intra-procedural monitoring.** Leaks in the inter-atrial baffles may occur along any part of the surgical suture lines. Closure of baffle leaks typically requires a device adaptable to various locations [44]. Currently, ASD occluders are the most commonly used devices [44], even though a VSD occluder may also be adopted. However, in cases of concurrent baffle stenosis, covered stent implantation is a viable alternative [45].

As with ASD closure, addressing baffle leaks requires balloon stretching to determine the appropriate device size. Static balloon interrogation provides echocardiographic and fluoroscopic measurements of the “stretched” leak. During this process, TOE is essential for confirming effective and complete leak closure, ensuring that there are no residual shunts. The presence of a “peri-balloon” residual shunt may indicate underestimation of the stretched diameter, warranting further balloon inflation. In certain cases, a leak balloon occlusion test can be performed to predict the hemodynamic response to closure. During this test, TOE is crucial for verifying complete leak closure to avoid erroneous hemodynamic data.

**Figure 16:**
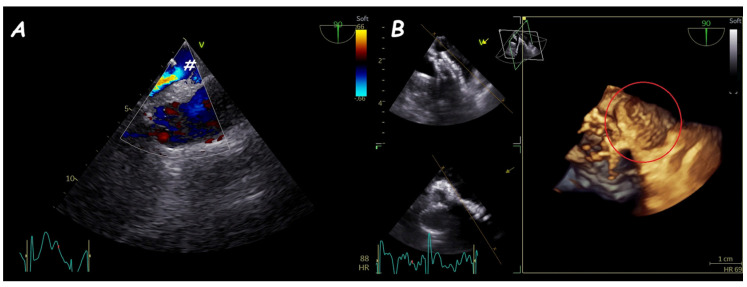
Two−dimensional TOE. Mid−oesophageal view of systemic baffle leak (#) with left−to−right shunt of a patient with TGA who has undergone a Mustard procedure (**A**). Three−dimensional TOE. Percutaneous closure of the baffle leak with an atrial septal defect occluder (circle) (**B**).

After device deployment, TOE should confirm proper disc “conformation” (i.e., absence of cobra or stretched shapes) and ensure that there are no interferences with peri-baffle structures (Figure 16). Intra-device residual shunts are common but typically resolve following device endothelization. Conversely, peri-device residual shunts are less frequent and may result from an adjacent fenestration (requiring an additional device) or incomplete coverage of the leak (necessitating a larger device). When multiple leaks are present, careful planning is required to achieve complete closure with the fewest devices. In such cases, the use of a covered stent graft is often the most effective solution for complete closure of all shunts.

## 5. Fontan Conduit Fenestration Management

**Pre-operative assessment.** Total cavo-pulmonary connection (Fontan shunt) represents the final palliative step in the management of functional univentricular hearts. Currently, the extracardiac conduit is the most commonly used surgical technique. In high-risk patients, a fenestrated conduit may be employed to create a small shunt between the Fontan circuit (systemic venous and pulmonary circulation) and the atrium, aiming to increase cardiac output in the early postoperative period. However, a patent fenestration can result in a right-to-left shunt, causing significant cyanosis. In such cases, percutaneous closure may be considered. Additionally, in lateral tunnel Fontan repairs, a residual right-to-left shunt due to suture dehiscence can lead to cyanosis, indicating the need for percutaneous closure.

Percutaneous closure of an extracardiac Fontan fenestration is primarily a fluoroscopy-guided procedure; however, TOE provides crucial support by offering a comprehensive view of the fenestration. TOE is essential for assessing the fenestration’s features, including size, shunt characteristics, and its relationship with intra- and extra-cardiac structures. Fenestrations are typically circular, although oval or irregular shapes are also observed. Three-dimensional TOE offers optimal visualization of the fenestration’s shape and dimensions [46]. In cases of lateral tunnel Fontan leaks, TOE is often indispensable for detecting right-to-left shunts and guiding the procedure (e.g., choice of venous approach—jugular vs. femoral—guidewire, catheter, and device) [47].

**Intra-procedural monitoring.** The most common techniques for closing a patent extracardiac Fontan fenestration include the implantation of an occluder device or the deployment of a covered stent. A balloon occlusion test is strongly recommended to evaluate hemodynamic changes following fenestration closure. After 10–15 min of balloon occlusion, a central venous pressure exceeding 18 mmHg, a central venous saturation reduction > 10%, or an arterial oxygen saturation increase < 95% are considered contraindications for fenestration closure. During the balloon occlusion test, TOE is essential to confirm complete closure of the fenestration and to exclude the presence of peri-balloon residual shunts.

**Figure 17:**
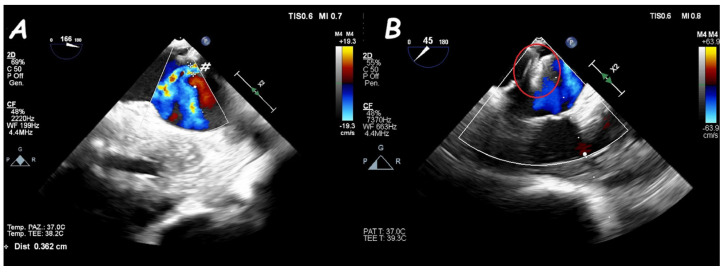
Two−dimensional TOE. Fenestration of an extracardiac Fontan conduit with right-to-left shunt (#) (**A**) that has undergone percutaneous closure with an atrial septal defect occluder (circle) (**B**).

For device closure, TOE is invaluable in monitoring device deployment, ensuring no interference between the device discs, intra-cardiac structures, and the Fontan conduit (Figure 17). In some cases, the right disc of the device may interfere with blood flow within the Fontan conduit. When such interference occurs, a smaller device or a covered stent implantation is preferred. After releasing the device, TOE must confirm complete closure of the fenestration (without intra- or peri-prosthetic shunt) and verify that there is no interference with the Fontan conduit or surrounding intra-cardiac structures.

In cases of lateral tunnel Fontan leaks, device deployment is the preferred strategy for closing the right-to-left shunt, though Uberoi et al. described successful closure using a self-expanding stent graft [48]. When multiple shunts are present, an attempt should be made to close all shunts. Often, a single occluder placed in the largest and/or central shunt achieves complete closure, eliminating the need for multiple devices. However, when shunts are widely spaced, two or more devices may be necessary.

TOE plays a critical role in identifying potential complications during the procedure, such as occluder disc thrombosis, peri-occluder damage, cardiac tamponade, device malposition, or embolization.

In certain cases, patients with Fontan circuit failure may benefit from the creation of a new fenestration (“ex novo” fenestration). TOE is vital in identifying the optimal site for conduit puncture and assessing the adequacy of the resulting shunt. Modern devices like the Atrial Flow Regulator (Occlutech International AB, Helsingborg, Sweden) facilitate the creation of a long-lasting shunt [49].

## 6. Ruptured Sinus of Valsalva Closure

**Pre-operative assessment.** Sinus of Valsalva aneurysms (SVAs) are characterized by significant dilatation of one or more sinuses of the aortic root. These aneurysms may be unruptured or ruptured, the latter typically resulting in a cardiac chamber shunt. In recent years, trans-catheter approaches have become feasible for closing ruptured SVAs. Sakakibara et al. [50] classified ruptured SVAs into four types:

- Type I, between the right coronary sinus and the distal right ventricle outflow tract (RVOT);
- Type II, between the right coronary sinus and the proximal RVOT;
- Type III, between the right coronary sinus and either the inlet of the right ventricle (IIIb or IIIv) or the right atrium (IIIa);
- Type IV, between the no-coronary sinus and the right atrium.

A modified Sakakibara classification includes a type V ruptured SVA, which represents rare cases where the aneurysm ruptures into the left atrium, pulmonary artery, left ventricle, or other chambers [51].

The “windsock sign” is a characteristic echocardiographic feature indicative of a ruptured SVA. Ruptured SVAs present with varying shapes (window-like, aneurysmal, or tubular) and sizes [52]. Identifying the “SVA type” is crucial for procedural planning, as it determines the approach (retrograde vs. anterograde). Additionally, the aneurysm’s shape and size—specifically, the size of the aneurysmal “sac” and the diameter of the fissured wall—are critical for selecting the appropriate closure device [52]. Single-disc cuneiform devices, often used for arterial duct occlusion, are preferred for window-like and aneurysmal SVAs, while double-disc vascular plugs are more suitable for tubular aneurysms [52].

Any associated right-, left-, or bi-ventricular dilatation or dysfunction should be assessed pre-operatively. Additionally, concomitant lesions, such as a bicuspid aortic valve, ascending aortic dilatation, or ventricular septal defects, should be evaluated, as they are frequently observed [53].

**Figure 18:**
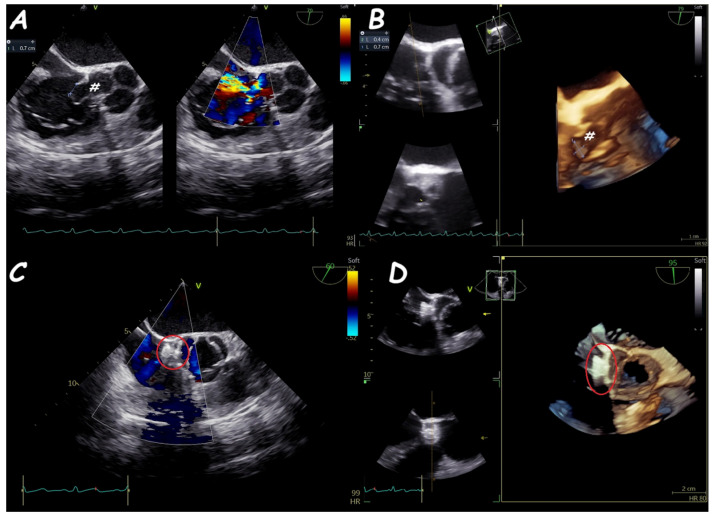
Trans−catheter closure of ruptured SVA. Two−dimensional (**A**) and three−dimensional (**B**) views of a ruptured aneurysm of the right sinus of Valsalva (#) with the characteristic “windsock sign.” Two−dimensional (**C**) and three−dimensional (**D**) views of a single disc device closure (i.e., duct occluder device) without residual shunt and interference with aortic valve function.

**Intra-procedural monitoring.** Trans-catheter closure of ruptured SVAs relies heavily on TOE to monitor device deployment. TOE facilitates precise tracking and navigation of wires, catheters, and devices, especially during the creation of the arterial-venous circuit. Once the device is implanted, it must achieve complete closure of the SVA shunt. While intra-prosthetic shunts are commonly observed and typically resolve, peri-prosthetic shunts should not be present. Furthermore, the device must not interfere with aortic valve function or coronary ostia (Figure 18).

In the trans-catheter closure of the ruptured SVAs, the TOE is crucial in monitoring the device deployment. The TOE allows accurate and effective tracking and navigation of wires, catheters, and devices (above all during the building of the arterial-venous circuit). After the implantation, the device should be placed to have complete closure of the SVA shunt (an intra-prosthetic shunt is usually detected, whereas a peri-prosthetic shunt should not be present), and it should not interfere with the aortic valve function or coronary ostia (Figure 18). In type I and III ruptured SVAs, it is essential to rule out device interference with the pulmonary and tricuspid valves, respectively [54].

Rare procedural adverse events are device thrombosis, device embolization, cardiac perforation, valvular interferences (stenosis, regurgitation, or cusps/leaflets perforation), coronary embolism or impingement, and conduction abnormalities (complete atrioventricular block) [55].

## 7. Miscellaneous

Percutaneous pulmonary valve replacement is the preferred treatment for significant pulmonary stenosis and/or regurgitation in patients with repaired CHDs. However, the procedure carries a risk of tricuspid valve damage, including leaflet injury or chordal rupture [56]. Intraprocedural TOE is useful for monitoring tricuspid valve integrity and avoiding damage.

Recent advancements include the adoption of percutaneous closure for superior vena cava ASDs in select anatomies. Although primarily guided by fluoroscopy, TOE is used to confirm complete closure of the ASD after stent deployment and to prevent significant iatrogenic stenosis of the right pulmonary veins [57].

Trans-catheter closure of baffle leaks following Takeuchi repair of anomalous left coronary artery from the pulmonary artery is another feasible option, with TOE providing critical monitoring to avoid interference with “peri-baffle” structures [58,59].

Procedures involving trans-septal puncture, such as left atrial appendage closure, pulmonary vein angioplasty or stenting, and mitral valve leak closure, require TOE guidance. TOE ensures optimal inter-atrial septum crossing and minimizes procedural risks [60].

Innovations in 3D ultrasound and virtual reality modeling now allow for simulations of device deployment in patients with complex anatomies [61]. These tools enhance procedural planning and reduce the risk of failure or complications. Advancements in ultrasound science, such as Transformative Noise Reduction (TNR) [62] and Self-Supervised Learning (SSL) [63], have the potential to automate and refine TOE image analysis. These technologies could lead to more accurate measurements and improved procedural guidance.

## Figures and Tables

**Table 1:** Pre-procedural assessment algorithm in trans-catheter ASD closure.

ASD Closure: Pre-Procedural Assessment	
ASD shape	elliptical, round or irregular shapes (star-, reniform- or other irregular shapes), internal seedings
ASD size	maximum ASD diameter
ASD shunt direction	left-to-right, bidirectional, or right-to-left
ASD periorificial rims	adequate (>5 mm), poor (<5 mm), or absent
Accessory ASDs	numbers, size, and distance (if present)
Atrial septum aneurysm	base width and an aneurysm excursion into the right or left atrium (if present)
Misaligned ASD	distance of separation between the septum primum surface and the septum secundum one (if present)
Double atrial septum	separation between the left atrial and right atrial rims of the defects (if present)
Redundant Eustachian valve	length and excursion (if present)
Chiari network	width and extension (if present)

**Table 2:** Pre-procedural assessment algorithm in trans-catheter VSD closure.

VSD Closure: Pre-Procedural Assessment	
VSD topography	peri-membranous or muscular VSD (inlet, outlet, or trabecular VSD; doubly committed VSD; malalignment VSD; Gerbode VSD)
VSD shunt direction	left-to-right, bidirectional, or right-to-left
VSD shape	elliptical, circular, or irregular shapes
VSD size	maximum ASD diameter
VSD periorificial rims	adequate (>2 mm), poor (<2 mm), or absent
Accessory VSDs	numbers, size, and distance (if present)
VSD aneurysmal pouch	base width and aneurysmal depth

## Data Availability

Not applicable.
